# Dangerous liaisons: interplay between SWI/SNF, NuRD, and Polycomb in chromatin regulation and cancer

**DOI:** 10.1101/gad.326066.119

**Published:** 2019-08-01

**Authors:** Adrian P. Bracken, Gerard L. Brien, C. Peter Verrijzer

**Affiliations:** 1 Smurfit Institute of Genetics, Trinity College Dublin, Dublin 2, Ireland;; 2 Department of Biochemistry, Erasmus University Medical Center, 3000 DR Rotterdam, the Netherlands

**Keywords:** NuRD, Polycomb, SWI/SNF, cancer, chromatin

## Abstract

Changes in chromatin structure mediated by ATP-dependent nucleosome remodelers and histone modifying enzymes are integral to the process of gene regulation. Here, we review the roles of the SWI/SNF (switch/sucrose nonfermenting) and NuRD (nucleosome remodeling and deacetylase) and the Polycomb system in chromatin regulation and cancer. First, we discuss the basic molecular mechanism of nucleosome remodeling, and how this controls gene transcription. Next, we provide an overview of the functional organization and biochemical activities of SWI/SNF, NuRD, and Polycomb complexes. We describe how, in metazoans, the balance of these activities is central to the proper regulation of gene expression and cellular identity during development. Whereas SWI/SNF counteracts Polycomb, NuRD facilitates Polycomb repression on chromatin. Finally, we discuss how disruptions of this regulatory equilibrium contribute to oncogenesis, and how new insights into the biological functions of remodelers and Polycombs are opening avenues for therapeutic interventions on a broad range of cancer types.

## Abstract (precis)

In this review, Bracken et al. discuss the functional organization and biochemical activities of remodelers and Polycomb and explore how they work together to control cell differentiation and the maintenance of cell identity. They also discuss how mutations in the genes encoding these various chromatin regulators contribute to oncogenesis by disrupting the chromatin equilibrium.

Chromatin is fundamental to all processes involving the eukaryotic genome. The nucleosome—147 bp of DNA wrapped tightly in ∼1.7 left-handed superhelical turns around an octamer of histones H2A, H2B, H3, and H4—is the fundamental repeating unit of chromatin. The need to compact genomic DNA (∼2 m for the human genome) to fit into the cellular nucleus (with a diameter of only ∼10 µm) is often presented as the rationale for nucleosomes. However, the packing fraction of DNA within the nucleus of a somatic cell is typically only about 1%, leaving ample unoccupied space. Therefore, rather than solving a physical packaging problem, nucleosomes instead provide a functional organization of the genome, enabling regulation of its replication, repair, and transcription. In fact, the most pertinent consequence of packaging genomic DNA into chromatin is that nucleosomes can impede access of DNA-binding proteins, such as transcription factors. Consequently, chromatin remodeling constitutes a fundamental level of gene expression control.

Central to chromatin organization, ATP-dependent chromatin remodeling enzymes (remodelers) are molecular motors dedicated to the assembly, positioning, or disruption of nucleosomes (Becker and Workman 2013; Clapier et al. 2017). By modulating the presentation of DNA, chromatin remodelers provide a fundamental level of gene expression control. In addition, chromatin state is regulated through a plethora of posttranslational modifications, in particular of the unstructured N-terminal histone tails that protrude from the nucleosome (Zentner and Henikoff 2013; Allis and Jenuwein 2016). These modifications, when present at specific residues on the histone N-terminal tails, can promote or antagonize the recruitment of regulatory proteins and may directly affect the compaction of the chromatin fiber. The local pattern of histone modifications is closely correlated with the transcriptional state of the associated gene or regulatory DNA element. For example, histone acetylation is generally associated with active chromatin irrespective of which residue is modified. In contrast, for histone methylation, the specific residue that is modified determines whether it is an active or a repressive mark. For example, while methylation of histone H3 at Lys4 (H3K4) by the MLL/COMPASS methyltransferases is associated with active transcription (Piunti and Shilatifard 2016), trimethylation at Lys27 (H3K27me3) is central to gene silencing by the Polycomb system (Schuettengruber et al. 2017). Although remodelers and histone-modifying enzymes, such as members of the Polycomb group, catalyze fundamentally different biochemical reactions, they function in an integrated manner to determine chromatin state. Here, we review how remodelers and Polycombs modulate the chromatin template to regulate gene expression. We also examine the interplay between the SWI/SNF (switch/sucrose nonfermenting) and NuRD (nucleosome remodeling and deacetylase) remodelers with Polycombs in human cancer and how our expanding understanding of their biology is guiding the development of new cancer treatments.

## ATP-dependent DNA translocation drives nucleosome remodeling

**Figure 1.**
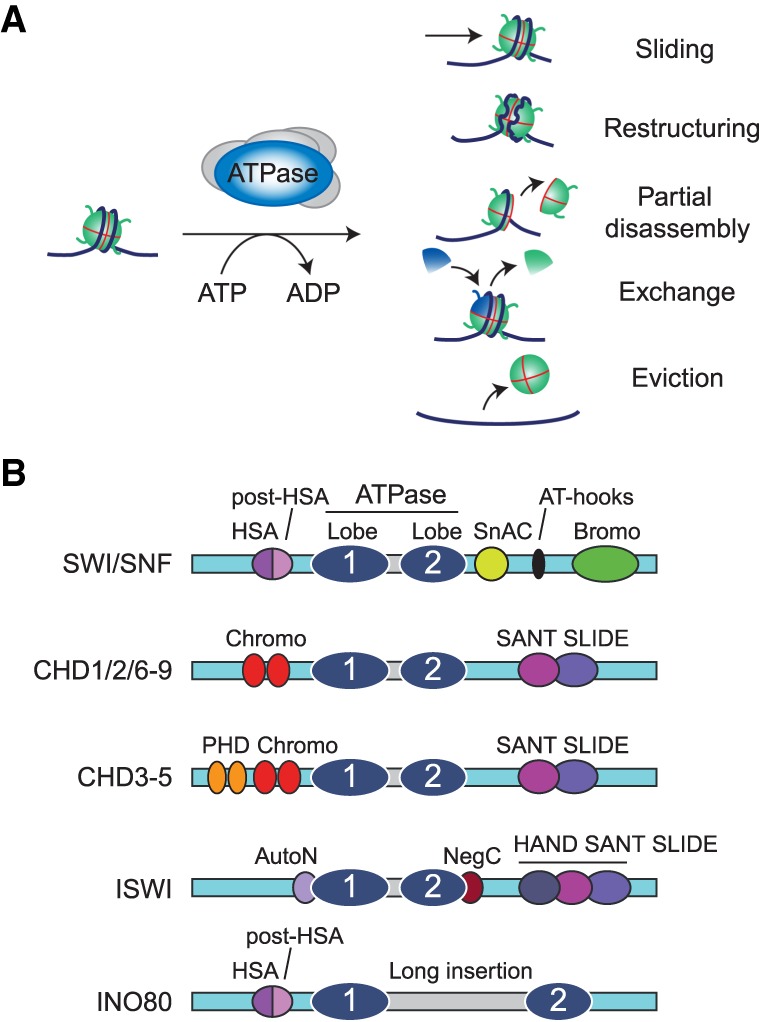
ATP-dependent chromatin remodeling. (*A*) Different outcomes of ATP-dependent remodeling of nucleosomes. Remodeler action can drive the sliding of a nucleosome to another position on the DNA, thus exposing a previously bound sequence. Alternatively, remodelers can make the nucleosomal DNA more accessible, while the histone octamer remains associated. Remodeling can also disrupt the octamer structure causing a partial disassembly, typically through eviction of histone H2A/H2B dimers. Specialized remodelers can mediate the exchange between histone variants. Finally, remodeling can result in the complete eviction of the histone octamer. (*B*) Structural domains of the four major Snf2 ATPase subfamilies, SWI/SNF, CHD, ISWI, and INO80. The translocase/ATPase domain of all remodelers comprises two RecA-like lobes separated by an insertion (highlighted in gray). Members of the INO80 family have a longer insertion than other remodelers. Each subfamily is characterized by a unique set of additional domains, including the HSA (helicase SANT-associated) and post-HSA domains, SnAC (Snf2 ATP coupling), AT hooks (A/T-rich DNA-binding domains), Bromo (bromodomains), Chromo (chromodomains), SANT-SLIDE domain, PHD finger (plant homeodomain), HAND-SANT-SLIDE domain, AutoN (autoinhibitory N-terminal), and NegC (negative regulator of coupling). See the text for details and references.

To understand how chromatin remodelers are powerful regulators of gene transcription, it is first necessary to understand their mechanisms of action. A chromatin remodeling reaction can have a variety of different outcomes (Becker and Workman 2013; Clapier et al. 2017). Through a sliding mechanism, a remodeler can move a nucleosome along the DNA template (Fig. 1A). Remodelers can generate a poorly understood remodeled state, in which the DNA becomes more accessible, but the histone octamer does not translocate to a new position. The action of remodelers can also result in a partial disruption of the nucleosome structure (e.g., through the eviction of a histone H2A/H2B dimer), while some remodelers mediate the exchange between histone variants. Finally, remodeling can lead to the complete eviction of the histone octamer. Whereas there are compelling examples of each of these mechanisms, their relative importance in vivo remains unclear. There are four major families of remodelers named after their central ATPase: SWI/SNF, INO80, ISWI, and CHD (Fig. 1B; Becker and Workman 2013; Clapier et al. 2017). Remodelers are further defined by unique sets of associated proteins that can modulate their activity or recruitment to chromatin. The various remodelers perform a wide-range of mostly nonredundant functions in the maintenance, transmission, and expression of eukaryotic genomes (Becker and Workman 2013; Clapier et al. 2017). For example, CHD1 and the ISWI class ACF remodelers mediate the formation of regular nucleosomal arrays, whereas SWI/SNF mediates their local disruption. INO80 class remodelers catalyze the exchange between the canonical histone H2A and the variant H2A.Z in nucleosomes.

**Figure 2.**
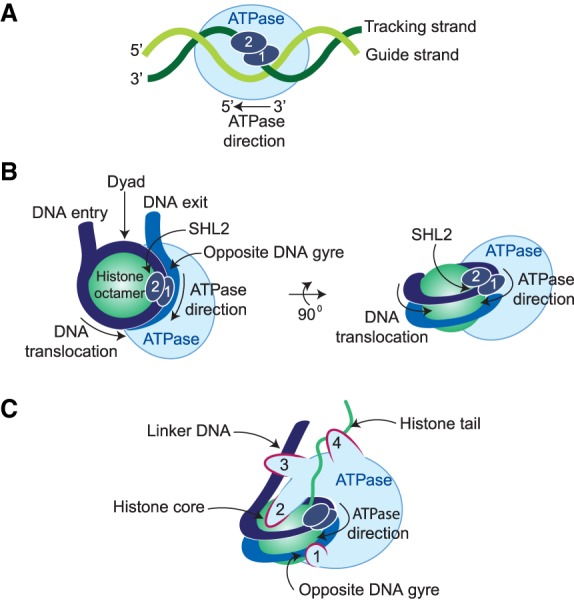
Model of nucleosome remodeling. (*A*) Cartoon of a generic ATP-dependent translocase RecA lobe 1 and lobe 2 moving along the tracking strand in a 3′ to 5′ direction. A cycle of ATP-binding and hydrolysis drives conformational changes through which the translocase “inchworms” along the tracking strand with 1-bp steps per every ATP hydrolysis. (*B*) Top and side view of remodeler ATPase binding to a nucleosome. The ATPase subunits of SWI/SNF, ISWI, CHD1, and SWR1 bind the nucleosomal DNA at superhelical position 2 (SHL + 2). (*C*) Remodeler ATPases make additional contacts through (1) binding to the opposite DNA gyre, ∼90 bp away; (2) contacting the histone core, typically at an acidic patch formed by H2A and H2B; (3) binding the linker DNA; and (4) interacting with the N-terminal tail of (usually) histone H4. Due to these additional contacts, the ATPase does not move along the nucleosomal DNA but rather pulls the DNA toward the octamer dyad. Multiple cycles of ATP-binding and hydrolysis generates a ratcheting motion that locally distorts the DNA and peels it off the histone core. See the text for details and references.

The basic action of the ATPase in different remodeling complexes appears to be largely similar (Clapier et al. 2017). Here, we highlight the salient aspects of our current understanding of remodeler function, in particular those relevant for SWI/SNF and NuRD. All remodelers contain a single motor subunit that belongs to the superfamily of ATP-dependent DNA and RNA translocases and helicases. The ATPase domain within the catalytic subunit is split into two domains with homology to the ATPase domain of the *Escherichia coli* RecA DNA-binding protein, referred to as lobes 1 and 2. The catalytic subunits of remodelers contain class-specific domains that can modulate their activity or mediate binding to DNA or histones (Fig. 1B). The noncatalytic subunits of remodeler complexes provide a plethora of additional functionalities, including regulation of the ATPase, providing contacts with DNA, histones, histone chaperones or sequence-specific transcription factors. A large body of studies on the mechanisms and structures of remodelers engaged with nucleosomes suggest a common mode of action. Fundamental to chromatin remodeling is the ATP-dependent translocation of DNA along the histone core of the nucleosome (Saha et al. 2002; Whitehouse et al. 2003; Clapier et al. 2017). Studies on classic translocases revealed that they move along one of the DNA strands, named the tracking strand, while the other strand is referred to as the guide strand (Fig. 2A). The ATPases of SWI/SNF, ISWI, and CHD1 all bind to the nucleosomal DNA at superhelical position 2 (SHL + 2), which is located two helical turns away from the nucleosomal dyad (Fig. 2B; Farnung et al. 2017; Liu et al. 2017; Sundaramoorthy et al. 2018; Li et al., 2019; Yan et al., 2019). In the resting state, the two lobes have an open conformation, separating the Walker A and B motifs in lobe 1 from a crucial arginine in lobe 2. ATP binding is accompanied by a conformational change (referred to as “closed state”) that creates a binding pocket comprising the Walker A and B motifs in lobe 1 and the catalytic arginine in lobe 2. Following ATP hydrolysis, the two lobes open up again, creating a cycle of ATP-binding and hydrolysis that drives movement of the translocase along the DNA tracking strand using an inchworm mechanism with a 1-bp step per ATP hydrolysis (Clapier et al. 2017; Li et al., 2019). However, nucleosome remodeling involves additional contacts between the remodeler and the nucleosome (Fig. 2C). These include binding of the ATPase to the opposite DNA gyre and the histone core. In particular, an acidic patch formed by histones H2A and H2B is frequently in physical contact with remodelers (Dann et al. 2017; Gamarra et al. 2018). Moreover, some remodelers interact with the N-terminal tail of histone H4 or contain additional DNA-binding domains that bind the linker DNA. Binding to these additional sites fixes the position of the translocase, preventing it from walking along the nucleosomal DNA. Instead, the remodeler will now pull the DNA toward the octamer dyad. This creates a ratcheting cycle in which the DNA is locally distorted, and through a combination of translational and rotational displacement peeled off the histone core (Clapier et al. 2017; Li et al., 2019). Throughout this remodeling process the histone core does not appear to undergo a major deformation (Yan et al. 2019). In summary, nucleosome remodeling depends on ATP-dependent DNA translocation driven by a motor domain that is fixed onto the nucleosome through additional DNA and histone contacts. Protein domains outside the RecA lobes play crucial roles in remodeler functionality (Clapier et al. 2017). Finally, in most remodelers, the ATPase activity is modulated by accessory subunits that determine remodeler function and targeting to specific genomic loci.

## How chromatin remodelers regulate transcription

**Figure 3.**
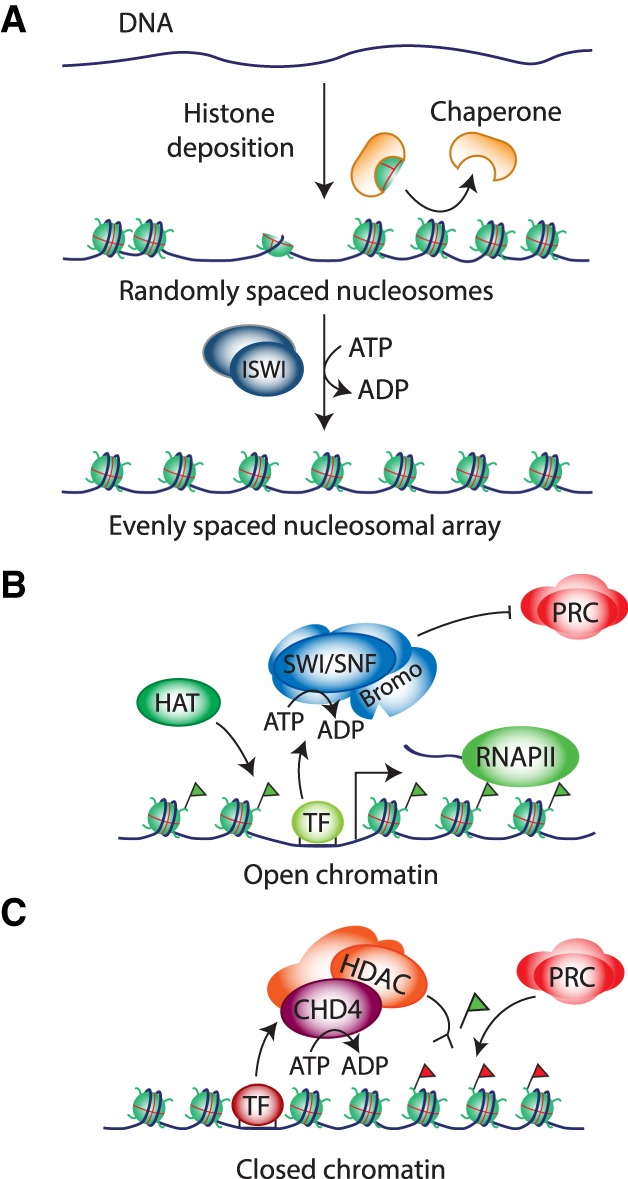
Remodeler functions in organizing the chromatin template. (*A*) Remodelers such as the ISWI class ACF mediate the formation of regularly spaced nucleosomal arrays; e.g., following DNA replication or other disruptions of chromatin organization. Well-organized arrays help prevent spurious initiation of transcription. (*B*) SWI/SNF remodelers promote transcription activation by generating an open chromatin conformation at promoters and enhancers, which may involve the sliding, displacement, or restructuring of nucleosomes. The relative importance of each of these mechanisms in vivo remains to be determined. Remodeler targeting involves recruitment by sequence-specific transcription factors and the local chromatin state; e.g., through recognition of acetylated histones by one of the bromodomains of SWI/SNF. In addition, SWI/SNF remodelers counteract Polycomb-repressive complexes (PRCs). (*C*) NuRD remodelers antagonize SWI/SNF function. NuRD mediates nucleosome invasion of regulatory DNA, and removal of acetylation marks. NuRD activity is then thought to promote the subsequent recruitment of the Polycomb system via its deacetylation of H3K27 and/or nucleosome remodelling, to further the formation of repressive chromatin. Green flags represent histone acetylation, and red flags represent H3K27me3.

At its most basic level, remodelers control gene transcription by mobilizing nucleosomes to make gene regulatory elements more or less accessible to the transcription machinery. Nucleosomes present a barrier for RNA polymerase II (RNAPII), and consequently there is no basal transcription on chromatin templates. Rather, gene transcription on chromatin requires sequence-specific transcription factors, which use coregulators, including remodelers, histone-modifying enzymes, and chaperones. Different remodelers perform diverse, nonredundant functions in the transcription cycle. Several ISWI class remodelers function in the assembly and generation of regularly spaced nucleosomal arrays (Fig. 3A). This plays a crucial role in the packaging of newly synthesized DNA following replication. Moreover, studies in yeast revealed that the generation of evenly spaced nucleosomes in gene bodies by ISWI and CHD1 remodelers helps to repress cryptic initiation of transcription (Becker and Workman 2013; Clapier et al. 2017). SWI/SNF remodelers have been implicated in generating an open chromatin conformation at gene promoters and enhancers (Fig. 3B). Induction of the unfolded protein response transcription program in *Drosophila* cells caused extensive changes in nucleosomal DNA accessibility, without accompanying changes in nucleosome occupancy (Mueller et al. 2017). Several studies suggested the presence of “fragile” nucleosomes, with DNA that is highly accessible, at regulated promoters (Lai and Pugh 2017). A recent study showed that these fragile nucleosomes are partially unwrapped RSC remodeling intermediates, which result from cooperation between RSC and general regulatory transcription factors (Brahma and Henikoff, 2019). In addition to changing DNA accessibility through remodeling, remodelers can also affect the composition of the histone core. For example, the INO80 class remodelers mediate the replacement of canonical histone H2A by the H2A.Z variant (Clapier et al. 2017; Lai and Pugh 2017). H2A.Z containing nucleosomes are enriched around transcription start sites and increase accessibility of nucleosomal DNA.

A key mechanism of gene selectivity is the cooperation between remodelers and sequence-specific DNA-binding transcription factors. Remodeler recruitment can be re-enforced by local chromatin changes. Histone acetylation can promote the recruitment of SWI/SNF to specific loci through recognition of acetylated histones by a bromo domain (Becker and Workman 2013). As discussed below, SWI/SNF and the Polycomb repressors function antagonistically on many regulatory DNA elements. The NuRD complexes can oppose SWI/SNF at shared regulatory elements (Fig. 3C). NuRD can generate repressive chromatin through nucleosome placement at regulatory DNA elements and histone deacetylation. The Polycomb system might further advance the formation of a repressive chromatin environment through H3K27 methylation and chromatin compaction (discussed below). It must be stressed that the behavior of a substantial proportion of promoters does not conform to generalizations derived from averaging results from genome-wide studies, which should be considered more as rules of thumb than as dogmas that apply to all genes.

## Structural and functional diversification of SWI/SNF remodelers

The large multisubunit SWI/SNF complex was the first remodeler described and remains the best studied. SWI/SNF was originally identified genetically in *Saccharomyces cerevisiae* through screens for genes that were involved in expression of the HO nuclease, required for mating type switching (SWI) (Stern et al. 1984), and expression of the *SUC2* invertase, required for sucrose fermentation (SNF) (Neigeborn and Carlson 1984; Abrams et al. 1986). Several of the encoded proteins, including the Snf2 ATPase, turned out to reside in a common complex, named SWI/SNF (Becker and Workman 2013; Clapier et al. 2017). The observation that mutations in histones alleviated the requirement for SWI/SNF and changes in chromatin structure in *snf2* mutants, suggested that SWI/SNF functions through targeting chromatin (Winston and Carlson 1992; Kruger et al. 1995). Indeed, in vitro biochemical analysis of yeast SWI/SNF revealed ATP-dependent chromatin remodeling and increased DNA accessibility (Cote et al. 1994). These results supported a scenario in which SWI/SNF activity opens-up promoter chromatin to promote transcriptional activation. Following the discovery of yeast SWI/SNF, related complexes were identified in mammalian cells that performed similar chromatin remodeling and gene regulatory functions (Imbalzano et al. 1994; Kwon et al. 1994).

**Figure 4.**
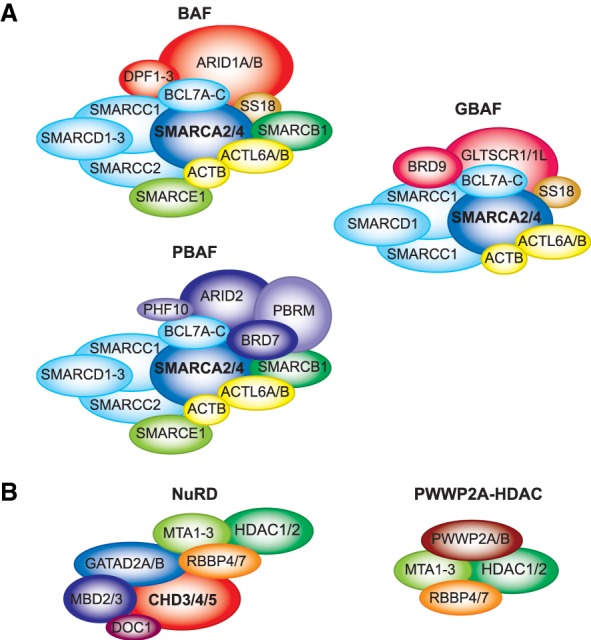
Composition of mammalian SWI/SNF and NuRD complexes. (*A*) Schematic representation of mammalian SWI/SNF complexes BAF, PBAF, and GBAF. Due to gene duplication events, several components of each complex are encoded by up to three paralogous genes in mammals. Alternative names and orthologous subunits in yeast and *Drosophila* are in Table 1. (*B*) Mammalian NuRD complex. A NuRD-related HDAC module lacking CHD3-5 and MBD2/3, associated with PWWP2A/B, is also illustrated. For details and references, see the text.

There are two main subtypes of SWI/SNF complexes that are broadly conserved among eukaryotes (Fig. 4A; Table 1). The first includes yeast SWI/SNF, *Drosophila* BAP, and mammalian BAF, while the second class includes yeast RSC, fly PBAP, and mammalian PBAF (Mohrmann and Verrijzer 2005). The corresponding complexes contain a variable number of identical subunits and paralogs that form a common core, associated with a set of signature subunits that are unique to either SWI/SNF-BAF or RSC-PBAF. Sth1, the ATPase of RSC, is a paralog of Snf2, the motor subunit of SWI/SNF. RSC and SWI/SNF contain four additional paralogs and share three subunits. The remaining subunits are unique for each complex. Both complexes are involved in activation of RNAPII transcription of largely nonoverlapping sets of genes and have been implicated in different aspects of DNA repair. RSC is also involved in RNA polymerase III transcription and several nontranscriptional chromosomal functions throughout the cell cycle (Clapier et al. 2017). Human cells contain two distinct SWI/SNF ATPases, named SMARCA4/BRG1 and SMARCA2/hBRM, which are both equally related to yeast Swi2/Snf2 and Sth1. Either ATPase associates with about eight additional subunits to form a core complex shared by both BAF and PBAF. In addition, there are two sets of mutually exclusive signature subunits that associate with the common core to form either BAF or PBAF. Polybromo (PBRM1), BRD7, ARID2, and PHF10 are specific for PBAF, whereas ARID1A/B and DPF1/2/3 define BAF. The differential incorporation of an array of paralogous subunits further increases the functional diversity of (P)BAF complexes. The isolated SMARCA2/4 ATPases are capable of remodeling in vitro, albeit at a lower level than the whole SWI/SNF complex (Phelan et al. 1999). The association of the core subunits SMARCB1, SMARCC1, and SMARCC2 with SMARCA4 suffices to restore untargeted remodeling activity to the level of the full SWI/SNF complex. It is instructive to compare the roles of *Drosophila* BAP and PBAP, because they share an identical remodeling core (Table 1). Functional dissection of fly SWI/SNF revealed that the subunits comprising the core play key architectural or enzymatic roles, whereas the BAP- and PBAP-specific subunits determine most of the genomic targeting and functional selectivity (Moshkin et al. 2012). BAP and PBAP have shared functions but also unique effects on gene expression, development, and cell cycle progression (Mohrmann et al. 2004; Moshkin et al. 2007, 2012; Chalkley et al. 2008). Recent comprehensive analyses of yeast and human SWI/SNF complexes provided detailed insights into their assembly, architecture, and functional organization (Dutta et al. 2017; Mashtalir et al. 2018). Collectively, structure–function dissection of the SWI/SNF remodelers suggests that they could be considered holoenzymes, in which different modules provide different functionalities that direct the remodeling activity.

**Table 1.**
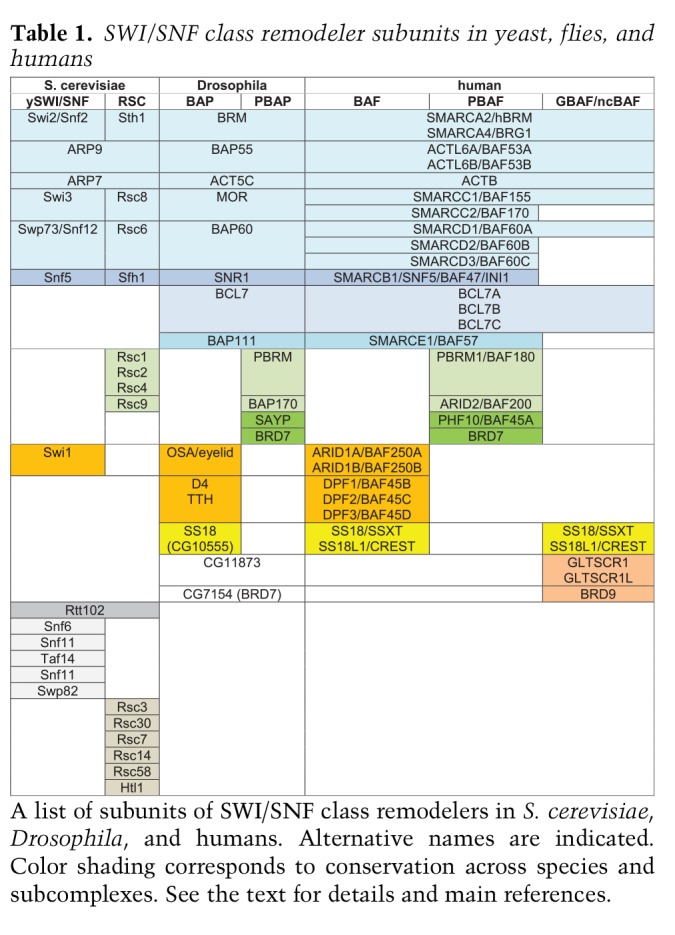
SWI/SNF class remodeler subunits in yeast, flies, and humans

A list of subunits of SWI/SNF class remodelers in *S. cerevisiae*, *Drosophila*, and humans. Alternative names are indicated. Color shading corresponds to conservation across species and subcomplexes. See the text for details and main references.

Recently, a third type of mammalian SWI/SNF complex was identified (Alpsoy and Dykhuizen 2018; Gatchalian et al. 2018; Mashtalir et al. 2018; Michel et al. 2018), named GBAF (glioma tumor suppressor candidate region gene 1 [GLTSCR1] BAF) or ncBAF (noncanonical BAF). GBAF/ncBAF comprises BRD9, GLTSCR1/1L, SMARCA2/4, ACTL6A/B, Actin, SMARCC1, SMARCD1, BCL7, and SS18/L1 (Fig. 4A; Table 1). Surprisingly GBAF lacks the conserved core subunit SMARCB1, which stimulates chromatin remodeling activity and genomic targeting of the canonical SWI/SNF complexes (Phelan et al. 1999; Kia et al., 2008; Nakayama et al. 2017; Sen et al. 2017; Wang et al. 2017). The presence of genes with homology to the GBAF-specific subunits in *Drosophila* raise the possibility that GBAF might be evolutionarily conserved. BRD9 plays a key role in directing GBAF to a specific set of genomic loci, in part through binding to BRD4 (Gatchalian et al. 2018; Michel et al. 2018). GBAF targets includes CTCF- and promoter-proximal sites (Michel et al. 2018), and in embryonic stem cells (ESCs), loci associated with naive pluripotency (Gatchalian et al. 2018). In summary, the SWI/SNF remodelers comprise a wide range of complexes that perform specialized, rather than generic functions.

## NuRD mediates nucleosome invasion and histone deacetylation

NuRD complexes bring together ATP-dependent chromatin remodeling and HDAC activities (Fig. 4B; Kolla et al. 2015; Torchy et al. 2015). Unlike SWI/SNF, which is present in all eukaryotes examined, NuRD remodelers, are restricted to metazoans. Mammalian NuRD complexes harbor one of three chromodomain ATP-dependent helicases (CHD3-5) and one of the histone deacetylases HDAC1 or HDAC2 (Kloet et al. 2015; Kolla et al. 2015; Torchy et al. 2015). CHD3-5 are unique amongst the CHD family in that they possess double PHD fingers in their N terminus (Fig. 1B). In addition, NuRD complexes contain one of two scaffolding proteins (GATAD2A/B), histone chaperones (RBBP4/7), one histone tail- and DNA-binding protein (MTA1–3), and one of the CpG-binding proteins (MBD2/3). Notably, MBD2, but not MBD3, has been proposed to preferentially bind methylated CpG residues (Menafra and Stunnenberg 2014). Alternatively, DNA methylation has been suggested to be required for the binding of both MBD2 and MBD3 (Hainer et al. 2016). Finally, the small DOC1 (deleted in oral cancer 1) protein is an integral subunit of all NuRD complexes and plays a role in its recruitment to target loci (Reddy et al. 2010; Spruijt et al. 2010; Mohd-Sarip et al. 2017). NuRD performs pivotal functions during development and stem cell differentiation (dos Santos et al. 2014).

NuRDs can act as transcriptional corepressors, which are recruited through sequence-specific transcription factors to induce robust gene silencing (Kehle et al. 1998; Reddy et al. 2010; Chudnovsky et al. 2014; Masuda et al. 2016; Liang et al. 2017). However, a global role for NuRDs in fine-tuning transcription has also been observed, in particular in ESCs (Günther et al. 2013; Shimbo et al. 2013; Bornelöv et al. 2018). Genome-wide studies revealed that CHD4 associates with the majority of promoters and enhancers in the mammalian genome, where it dampens the levels of cognate gene transcription (Whyte et al. 2012; Reynolds et al. 2012a; Günther et al. 2013; Shimbo et al. 2013; Bornelöv et al. 2018). Binding to the histone H3.3 variant might help to recruit NuRD to regions of active chromatin, where it then fine-tunes the level of transcription (Kraushaar et al. 2018). Temporal analysis of gene repression induced by the transcription factor Ikaros revealed that NuRD drives nucleosome invasion, RNAPII eviction, and reduced activator binding at target loci (Liang et al. 2017). This required chromatin remodeling by CHD4 but was independent of HDAC activity. Histone deacetylation occurs later and contributes to the maintenance of gene silencing. The use of a MBD3-inducible system in ESCs revealed wide association with active chromatin, and a role for MBD3-NuRD in transcriptional dampening that mainly involves nucleosome invasion (Bornelöv et al. 2018). Again, deacetylation of H3K27 followed attenuation of transcription, rather than preceding it. Thus, a model is emerging in which NuRD acts broadly at many enhancers and promoters to dampen and fine-tune active gene expression (Bornelöv et al. 2018). In summary, NuRD acts as a global modulator of transcription but can also function as a transcription factor recruited corepressor. These different modes of NuRD action might reflect a difference in the level of local NuRD recruitment by transcription factors versus a more general affinity of NuRD for open chromatin.

NuRD refers to a multitude of different protein assemblages. Consequently, studies based on a single subunit may only reflect the function of a particular subset of NuRD complexes. For example, MBD2-NuRD, rather than MBD3-NuRD, appears to form repressive chromatin (Günther et al. 2013; Menafra and Stunnenberg 2014). Whereas *Mbd3* KO mice are early embryonic lethal, *Mbd2* KO mice are viable (Hendrich et al. 2001; Kaji et al. 2006; Reynolds et al. 2012a). Therefore, caution must be taken not to generalize observations based on a particular subunit to all NuRD family complexes. The NuRDs appear to be more loosely assembled than SWI/SNF, and separation of remodeling- and HDAC modules has been reported (Kunert et al. 2009; Zhang et al. 2016, 2018; Link et al. 2018). These submodules may associate with selective partners that recruit them to distinct genomic loci. For example, a complex comprising the *Drosophila* Mi2 ATPase and the zinc-finger homeobox protein MEP1 has been identified (Kunert et al. 2009). A NuRD-related HDAC module, lacking CHD3-5 and MBD2/3, associates with PWWP2A/B, which recognizes the active chromatin features H2A.Z and H3K36me3 (Fig. 4B). Recruitment of this module by PWWP2A/B to active genes reduces the level of histone acetylation to decrease transcriptional elongation (Link et al. 2018; Zhang et al. 2018). Thus, some NuRD subunits are also part of alternate complexes that lack either remodeling or HDAC activity.

## Polycomb-repressive complexes (PRCs) in *Drosophila* and mammals

Polycomb group (PcG) proteins are a large family of conserved chromatin regulators that are essential for maintaining cellular identity in higher eukaryotes (Schuettengruber et al. 2017). *PcG* genes were discovered in *Drosophila* as repressors of homeotic (*Hox*) genes (Kassis et al. 2017; Schuettengruber et al. 2017). The maintenance of established patterns of *Hox* and other developmental gene expression requires the antagonistic activities of the PcG and Trithorax group (TrxG) proteins. While the PcG proteins maintain repression, the TrxG proteins contribute to sustaining active gene transcription. The names of many of the *PcG* genes were inspired by the phenotype of extra sex combs appearing on the second and third pair of legs of male flies, when normally these bristles only form on the first pair of legs. This distinctive phenotype is caused by the derepression of the *sex combs reduced* Hox gene and provided a powerful diagnostic for subsequent genetic screens to identify additional *PcG*- and *TrxG* genes (Kassis et al. 2017).

**Figure 5.**
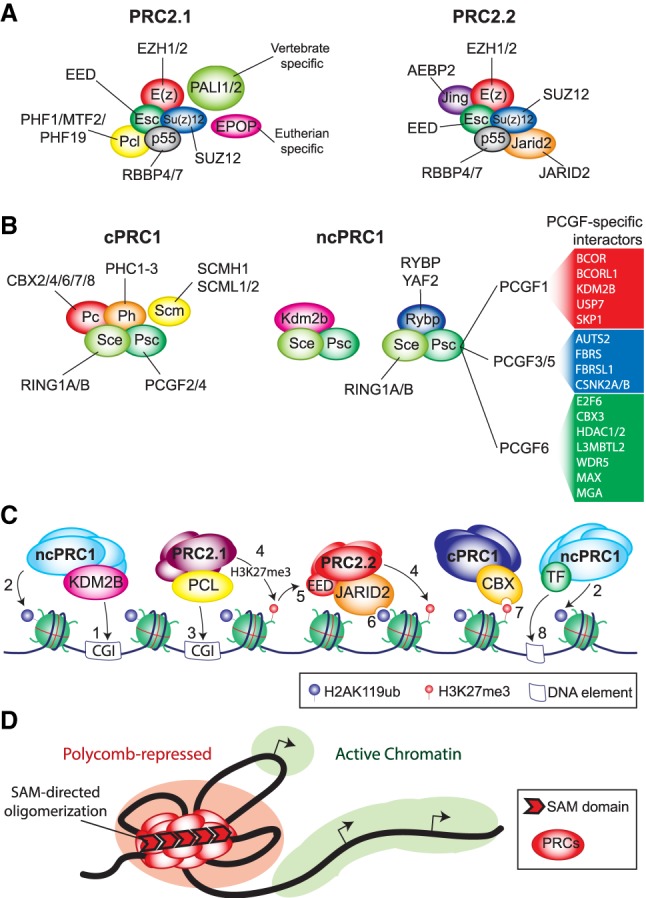
Polycomb group protein complexes and chromatin repression. (*A*) Schematic representation of PRC2.1 and PRC2.2 complexes. *Drosophila melanogaster* PRC2 components are shown in the colored ovals, and their mammalian homologs are also indicated. In mammals, PRC2 has a trimeric enzymatic core composed of EZH1/2–SUZ12–EED. PRC2.1 contains one PCL protein and the vertebrate- and eutherian-specific proteins PALI1/2 and EPOP, respectively. In PRC2.2, these proteins are replaced by AEBP2 and JARID2. (*B*) Schematic representation of cPRC1 and ncRC1. *D. melanogaster* PRC1 are indicated in the colored ovals. The names of their sometimes multiple mammalian homologs are also indicated. The enzymatic core of PRC1 is a RING-PCGF heterodimer (which is present in cPRC1) and ncPRC1. In cPRC1, RING-PCGF associates with one of each of the CBX, PHC, and SCM proteins. In ncPRC1, RING-PCGF associates with either a RYBP or YAF2 subunit. (*Right* panels) The different ncPRC1 complexes are defined by their specific PCGF subunit, which in turn associate with divergent subsets of interacting proteins. (*C*) Multiple ways of PRC recruitment to chromatin. As detailed in the text, KDM2B binds CpG islands (CGIs), thus targeting ncPRC1 (1) and H2A ubiquitylation (2). Polycomb-like proteins also bind CpG islands (3) and mediate H3K27 methylation by PRC2.1 (4). (5) H3K27me3 is recognized by EED, which then allosterically activates PRC2, thus facilitating the establishment of H3K27me3 domains. (6) The ncPRC1 mark H2Aub is recognized by JARID2, promoting local H3K27me3 by PRC2.2. (7) H3K27me3, in turn, is bound by CBX proteins in cPRC1 complexes that mediate chromatin compaction. (8) PCGF3/5/6 ncPRC1 complexes harbor sequence-specific DNA binding proteins that target H2Aub to chromatin. While, they also target nonclassical PcG sites, they may also contribute to promoting deposition of H3K27me3 via PRC2.2 binding to H2Aub. (*D*) Recruitment of the various PRC complexes generates a repressive chromatin environment characterized by H3K27me3, H2Aub, and chromatin compaction mediated by long-range interactions involving SAM domain-mediated polymerization of the CBX2 and PHC1-3 subunits. These PcG silenced domains are sometimes referred to as Polycomb bodies (red), that are separate in nuclear space from open transcribed chromatin (green).

In *Drosophila*, most PcG proteins function as part of two broad classes of multiprotein complexes, named PRC1 and PRC2 (Kassis et al. 2017; Schuettengruber et al. 2017). The core of *Drosophila* PRC2 is formed by E(z), Su(z)12, CAF1-p55, and Esc (Fig. 5A). E(z) is a SET domain containing histone methyltransferase that, as part of PRC2, trimethylates histone H3K27 (H3K27me3), which is essential for PcG repression (Pengelly et al. 2013). There are two main forms of PRC2 in flies, defined by whether they contain the alternative subunits Pcl (Polycomb-like) or Jarid2 (Nekrasov et al. 2007; Herz et al. 2012). The PRC1 class is further subdivided into canonical (cPRC1) and noncanonical PRC1 (ncPRC1) (Fig. 5B). *Drosophila* cPRC1 comprises Pc, Psc [or Su(z)2], Ph, and Sce (also known as dRing), and while Scm associates with cPRC1, it is considered a substoichometric subunit. cPRC1 binds PRC2-mediated H3K27me3 via the chromodomain of Pc (Cao et al. 2002; Fischle et al. 2003) and is thought to mediate repression through chromatin compaction and loop formation (Francis et al. 2004; Entrevan et al. 2016; Ogiyama et al. 2018). Fly ncPRC1, originally named dRAF (dRing-associated factors), is defined by a core complex composed of Sce, Psc, and Kdm2 but lacks Pc and Ph and can include Rybp (Lagarou et al. 2008; Fereres et al. 2014). ncPRC1 couples the removal of the active H3K36me2 mark with the deposition of H2AK118ub, whereas cPRC1 lacks appreciable H2A ubiquitylation activity (Lagarou et al. 2008). While the H2AK118ub mediated by ncPRC1 is required for viability and helps to promote PRC2 mediated H3K27me3, it is not essential for *Hox* gene repression (Kalb et al. 2014; Pengelly et al. 2015).

In mammals, PcG proteins perform essential functions during development, cell differentiation and disease (Schuettengruber et al. 2017). As in *Drosophila*, genome-wide binding studies in mammalian cells confirmed that PcG proteins directly bind to the gene loci of Hox and other key developmental regulators (Schuettengruber et al. 2007). Although the key PcG proteins, PRC organization, and histone modifications are all conserved, the Polycomb system has expanded in mammals, compared with flies (Schuettengruber et al. 2017). In mammals, both cPRC1 and ncPRC1 are defined by a heterodimeric RING-PCGF (PcG ring finger) core, which can function as an E3 ubiquitin ligase to monoubiquitinate H2AK119 (Lys118 in *Drosophila*) (Wang et al. 2004; McGinty et al. 2014; Blackledge et al. 2015). There are two variants of the RING subunit in mammals, RING1A and RING1B, each of which can form a heterodimer with one of six variants of the PCGF subunit PCGF1–6 (Fig. 5B; Gao et al. 2012). cPRC1 contains either PCGF2 (MEL18) or PCGF4 (BMI1) in addition to one chromobox (CBX2, CBX4, CBX6, CBX7, or CBX8), one sex combs midleg (SCMH1, SCML1, and SCML2), and one polyhomeotic (PHC1–3) subunit (Simon and Kingston 2009).

As in flies, the PRC2 complex is responsible for mediating all H3K27me1/2/3 on chromatin (Conway et al. 2015; Højfeldt et al. 2018). The core PRC2 complex is composed of one of the two histone H3K27 methyltransferases, Ezh1 or Ezh2, together with Suz12 and Eed, which are required for histone methytransferase activity (Margueron and Reinberg 2011). In addition, several accessory proteins associate with core PRC2, which are thought to modulate the recruitment and enzymatic activity of the complex (Fig. 5A; Laugesen et al. 2019). *Drosophila* Pcl has three mammalian homologs (Phf1, Mtf2, and Phf19), while Jarid2 and Aebp2 (Jing in flies) are conserved as well. Additional interacting proteins include Epop, Pali1, and Pali2 (Holoch and Margueron 2017; Conway et al. 2018). Comprehensive proteomic analyses showed that PRC2 primarily assembles into two mutually exclusive combinations, termed PRC2.1 and PRC2.2 (Alekseyenko et al. 2014; Hauri et al. 2016). PRC2.1 is defined as containing one of the three Pcl proteins, while PRC2.2 is defined as containing Aebp2 and Jarid2 (Margueron and Reinberg 2011; Holoch and Margueron 2017). There is additional variation within the PRC2.1 subtype, such that one Pcl protein is a constant and defining feature, while the presence of EPOP and PALI1 are mutually exclusive (Alekseyenko et al. 2014; Hauri et al. 2016). Therefore, while PRC2.1 and PRC2.2 are similar to *Drosophila* Pcl-PRC2 and Jarid2-PRC2, respectively, additional accessory proteins of PRC2.1 have emerged during evolution, providing additional opportunities for regulation (Fig. 5A).

## Mechanisms of recruitment and formation of Polycomb-repressive domains

The recruitment of PcG proteins has been well-studied in flies. It is mediated by specific *cis*-regulatory DNA sequences named Polycomb response elements (PREs) (Kassis et al. 2017). PREs are essential for the establishment and propagation of Polycomb-repressed chromatin (Busturia et al. 1997; Coleman and Struhl 2017; Laprell et al. 2017). The sequence-specific transcription factor Pho plays a central role in the recruitment of PRC1 and PRC2 to PREs (Brown et al. 1998; Frey et al. 2016; Erceg et al. 2017; Kassis et al. 2017). Pho and PRC1 bind to PREs cooperatively, generating a nucleosome free region (Mohd-Sarip et al. 2006, Schuettengruber et al. 2014). Pho associates with Sfmbt to form PhoRC (Klymenko et al. 2006). Next, Scm, through binding to Sfmbt, PRC1, and PRC2, provides a functional link between these three complexes (Kang et al. 2015; Frey et al. 2016). SAM-domain mediated polymerization of Scm and Ph might further contribute to the generation of PcG silenced domains or long-range interactions, possibly directed by H3K27me3 (Kang et al. 2015; Wani et al. 2016). While PHO-binding sites are necessary, they are not sufficient for PRC recruitment, consistent with the essential contributions of additional transcription factors (Kassis et al. 2017; Brown et al. 2018). In summary, PcG recruitment in flies is achieved through a network of protein–protein interactions and DNA-binding that tether PRCs to regulatory DNA elements. Rather than a universal hierarchical process, PcG repression involves a mix of cooperative and redundant mechanisms that differ in different contexts (Kassis et al. 2017; Brown et al. 2018; De et al. 2019).

In mammals, the role of PRC2-mediated H3K27me3 in directing cPRC1 to target genes via chromodomains within its CBX subunits (the mammalian Pc homologs) is well-defined (Margueron and Reinberg 2011). Indeed, the majority of H3K27me3 and PRC2-associated genes are cobound by cPRC1 complexes in multiple mammalian cell types (Boyer et al. 2006; Bracken et al. 2006). After binding, cPRC1 is thought to confer gene repression by chromatin compaction through its CBX and PHC1–3 subunits (Isono et al. 2013; Lau et al. 2017). While the role of H3K27me3 in directing cPRC1 is well-defined in mammalian cells, much less is known about the role of PRC2-mediated H3K27me2 (Conway et al. 2015). H3K27me2 is present on almost all intergenic euchromatin regions in both mammals and *Drosophila* (Ferrari et al. 2014; Conway et al. 2015; Lee et al. 2015) and has been proposed to form a repressive “genomic blanket” to prevent the misfiring of cell type-specific enhancers of alternative lineages (Conway et al. 2015). Thus, the PRC2 complex is believed to contribute to the maintenance of cellular identity via both H3K27me3 and H3K27me2.

As in *Drosophila*, ncPRC1 is responsible for the majority of H2A monoubiquitination in mammalian cells (Blackledge et al. 2014). The ncPRC1 assemblages all lack the CBX (Pc in flies) and PHC (Ph in flies) proteins (Gao et al. 2012). Instead, they contain one of the paralogs RYBP or YAF2 that interact directly with RING1A or RING1B. Another distinctive feature of mammalian ncPRC1 assemblages is that they contain any of PCGF1/3/5/6, each of which associates with specific additional subunits (Fig. 5B). For example, PCGF6-containing ncPRC1 harbors several transcription factors, including E2F6, MAX, and MGA, whereas the PCGF1-containing ncPRC1 includes the lysine demethylase KDM2B together with BCOR, SKP1, and the deubiquitylating enzyme USP7 (Fig. 5B). KDM2B can bind to unmethylated CpG islands via its CxxC motif and recruits PCGF1-ncPRC1, which can then deposit H2AK119ub (Fig. 5C; Wu et al., 2013; Blackledge et al. 2014). This H2AK119ub modification has been suggested to contribute to the recruitment of PRC2.2 via its Jarid2 subunit, which contains a ubiquitin-binding motif (Cooper et al. 2016). Supporting this model, reduced H2AK119ub in ESCs lacking either RING1 proteins or combinations of ncPRC1-specific PCGFs leads to a partial reduction in PRC2 and H3K27me3 levels on Polycomb target genes (Blackledge et al. 2014; Fursova et al. 2019; Scelfo et al. 2019). However, while the loss of RING1A/B in both mouse colon and skin cells in vivo leads to complete loss of H2AK119ub, global levels of H3K27me3 are maintained (Chiacchiera et al. 2016; Cohen et al. 2018). Therefore, it appears that while recognition of H2AK119ub by JARID2-PRC2.2 contributes to local accumulation of H3K27me3 in some contexts, it cannot be the sole mechanism. It is likely that even in the absence of H2A ubiquitylation, Polycomb-like proteins can still direct PRC2.1 to mediate H3K27me3. Supporting this, *Drosophila* embryos engineered to lack all H2AK118ub maintain the repression of *Hox* genes and do not exhibit a Polycomb phenotype (Pengelly et al. 2015). Furthermore, the loss of Pcl, but not Jarid2, leads to *Hox* gene derepression in *Drosophila*. Although the mechanisms still need to be worked out in mammalian cells, Polycomb-like proteins may provide alternate means of engagement with chromatin through binding to H3K36me2/3 and H3K27me3 via their conserved Tudor domain (Ballaré et al. 2012; Brien et al. 2012, 2015; Cai et al. 2013) or interaction with GC-rich DNA via their winged helix (WH) domain (Choi et al. 2017; Li et al. 2017; Perino et al. 2018).

In summary, it is clear that there are multiple interdependent interactions between the various PRCs that mediate their association with chromatin in mammalian cells (Fig. 5C). The ncPRC1 complexes can direct H2AK119ub to chromatin via either KDM2B binding to CpG islands or DNA-binding transcription factors. The H2AK119ub modification is in turn recognized by PRC2.2, which then deposits H3K27me3. PRC2.1 is targeted to chromatin via its Polycomb-like proteins. The combined action of both PRC2 subtypes results in a genomic profile of H3K27me3, which is then recognized by cPRC1. The activities of cPRC1 mediate long-range interactions and compact the chromatin via SAM-directed oligomerization, generating Polycomb-repressed domains (Fig. 5D). In conclusion, despite the general conservation of the Polycomb system in multicellular eukaryotes, expansion and subfunctionalization in vertebrates enables diverse mechanisms of recruitment and fine-tuning of gene expression.

## Regulatory interplay between NuRD, Polycomb, and SWI/SNF

The SWI/SNF, NuRD, and PcG complexes do not function in isolation but are part of a regulatory network that also involves other chromatin regulators and transcription factors. This was first highlighted by genetic studies in *Drosophila* that identified suppressors of PcG mutations, forming the TrxG genes (Kennison and Tamkun 1988; Kassis et al. 2017; Schuettengruber et al. 2017). The TrxG genes encode a diverse set of proteins involved in various aspects of chromatin regulation and transcription (Kassis et al. 2017; Schuettengruber et al. 2017). They include the MLL/COMPASS histone H3K4 methyltransferases (that includes Trx), subunits of the mediator complex, the cohesin subunit Rad21, the Brd4-related fs(1)h, the remodelers Brm and Kismet, and the sequence-specific DNA-binding proteins Gaga and Zeste. TrxG genes were identified in two different ways. Some, including the founding member *trx*, were identified as activators of *Hox* gene expression. Others, such as *Brm*, were discovered in screens for suppressors of *Pc* mutations (Kennison and Tamkun 1988; Kassis et al. 2017). *Brm* encodes the fly homolog of yeast Swi2/Snf2 (Table 1; Tamkun et al. 1992). The SWI/SNF core subunit Moira (Mor) and BAP-selective subunit Osa were also identified as suppressors of Pc (Kennison and Tamkun 1988; Collins et al. 1999; Crosby et al. 1999; Kal et al. 2000). Direct tests revealed that the PBAP signature subunits Bap170, Polybromo, and Sayp are also suppressors of Pc (Chalkley et al. 2008). The transcription factor Zeste belongs to the TrxG and has many binding sites within PREs. A combination of biochemical and in vivo observations showed that Zeste mediates the maintenance of an activated state through recruitment of (P)BAP (Kal et al. 2000; Déjardin and Cavalli 2004). Zeste binds to its DNA sites in a chromatin template by itself, but (P)BAP is required for transcription activation (Kal et al. 2000). Zeste tethers (P)BAP via direct binding to the TrxG subunits Mor (core), Osa (BAP) and Bap170 (PBAP). In conclusion, genetic studies in *Drosophila* established that SWI/SNF and Polycomb act antagonistically in a dosage-dependent manner. Analysis of the role of SWI/SNF in human cancer revealed the conservation of this mechanism of gene control. In rhabdoid tumor cells, the loss of SMARCB1 compromises SWI/SNF opposition of Polycomb repression (Kia et al. 2008; Wilson et al. 2010). Genome-wide analysis revealed that SWI/SNF counteracts Polycomb repression of a multitude of genes, in particular at bivalent promoters (Nakayama et al. 2017; Wang et al. 2017). Artificial recruitment of SWI/SNF leads to a rapid (within minutes), ATP-dependent eviction of PRCs, which is independent of RNAPII transcription (Kadoch et al. 2017; Stanton et al. 2017). These studies suggest that SWI/SNF can remove PRCs from the chromatin through a direct mechanism (Fig. 3B). Conversely, in vitro studies showed that PRC1 can inhibit chromatin remodeling by SWI/SNF (Francis et al. 2001). Collectively, these studies suggest that SWI/SNF and Polycomb compete in a dosage-dependend manner to generate opposing chromatin states.

NuRD and SWI/SNF also act antagonistically on common regulatory elements. In ESCs, SWI/SNF, and NuRD have opposite effects on the nucleosome organization of shared targets (Yildirim et al. 2011; Hainer et al. 2015). Likewise, in oral squamous carcinoma cells NuRD and SWI/SNF compete for binding to genes that encode master regulators of the epithelial-to-mesenchymal transition (EMT) (Mohd-Sarip et al. 2017). NuRD mediates the formation of repressive chromatin through nucleosome invasion, histone deacetylation, and subsequent Polycomb recruitment. In contrast, SWI/SNF generates open chromatin and counteracts Polycomb. These results suggest that transcriptional control involves a dynamic equilibrium between opposing chromatin modulating enzymes rather than a static chromatin state. In agreement with this notion, the induction of repressive chromatin in pre-B cells by the transcription factor Ikaros is accompanied by the replacement of SWI/SNF by NuRD (Liang et al. 2017). The *Drosophila* Ikaros-related transcription factor Hunchback represses the *Hox* genes early in development through NuRD recruitment (Kehle et al. 1998). After expression of Hunchback ceases later in development, gene repression is maintained by the Polycomb system. These early observations in *Drosophila* first suggested that NuRD might create a chromatin environment that facilitates subsequent Polycomb repression (Fig. 3C). This notion has been expanded by studies in mice, ESCs, and human cancer (Morey et al. 2008; Reynolds et al. 2012b; Egan et al. 2013; Sparmann et al. 2013; Mohd-Sarip et al. 2017). The loss of NuRD-mediated histone deacetylation activity in *Mbd3* null ESCs correlates with a loss of PRC2 association on a subset of CHD4 target genes (Reynolds et al. 2012b). Thus, deacetylation of H3K27 might be a prerequisite for methylation and subsequent stabilization of PRC2 binding (Fig. 3C). Likewise, the depletion of CHD5 leads to a loss of H3K27me3 on CHD5 and PRC2 cobound genes (Egan et al. 2013). The CHD3–5 proteins all contain two PHD and two chromodomains (Fig. 2A), which have been proposed to act synergistically to determine their histone-binding specificity (Egan et al. 2013). The double PHD domains bind to unmodified H3K4, while the double chromodomains bind to H3K27me3 in vitro (Egan et al. 2013; Paul et al. 2013). Thus, PRC2 might also help NuRD recruitment, creating a positive feedback loop for NuRD and PRC2 targeting to chromatin. Alternatively, through an indirect process, transcriptional repression by NuRD might allow the default binding of PRC2 to CpG islands of silenced genes (Riising et al. 2014). In conclusion, at least on a subset of regulatory sites, SWI/SNF and NuRD compete for access to chromatin and generate opposite chromatin states.

## Remodelers and Polycomb in human cancers

**Figure 6.**
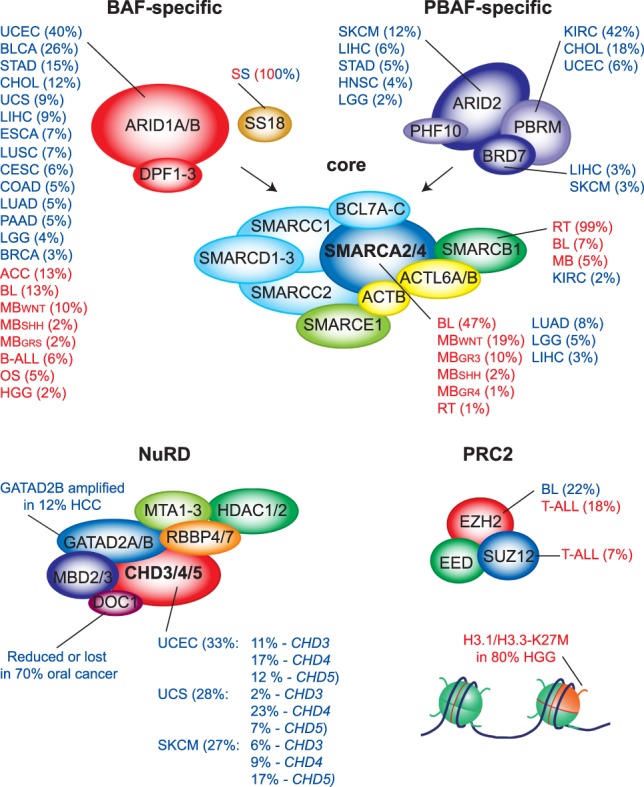
Chromatin remodelling complexes are frequently mutated in cancer. Mutations in different SWI/SNF subunits associate with different types of cancer. The percentage of mutations in specific types of cancer are indicated. Adult cancers are shown in blue, pediatric cancers in red. Cancer-associated mutations in NuRD do occur but are less frequent than in SWI/SNF. The enzymatic core of PRC2 is subject to both activating (in diffuse large B-cell and follicular lymphoma) and inactivating (in T-cell acute lymphoblastic leukemia and malignant peripheral nerve sheath tumors) mutations. The primary substrate of the PRC2 complex, H3K27, is also the target of an oncogenic mutation in the majority of pediatric diffuse intrinsic pontine glioma tumors. Mutation rates, which are indicated for each disease, are taken from the Cancer Genome Atlas (TCGA) pan-pediatric atlas (Gao et al. 2018) for adult cancers and for pediatric diseases from recent pan-pediatric cancer genmics studies (Gröbner et al. 2018; Ma et al. 2018). Cancer studies are indicted by their TCGA abbreviations. For details and references, see the text.

Cancer genome sequencing studies revealed that chromatin regulatory proteins are among the most highly mutated in human cancer (Bailey et al. 2018; Gröbner et al. 2018; Ma et al. 2018). This indicates that disruptions in the biochemical mechanisms that control chromatin dynamics may play a significant role in the development of many cancers. Approximately 30% of newly identified potential cancer “driver” mutations occur in genes that encode chromatin regulators. Within this functional class, genes encoding selective SWI/SNF subunits are remarkably prone to mutations (Kadoch et al. 2013; Shain and Pollack 2013; Masliah-Planchon et al. 2015). More than 20% of all cancers have mutations in SWI/SNF members, making these complexes the most frequently mutated remodelers in cancer (Fig. 6). Genes encoding different SWI/SNF subunits are mutated at high frequencies in specific, nonoverlapping malignancies, indicating that they might have nonredundant tumor-suppressive functions. In addition to clear truncating, loss-of-function or loss-of-expression mutations, a substantial portion of mutations in SWI/SNF genes are substitution mutations that are distributed rather evenly across the coding sequence at relatively low frequency. Although these substitutions might affect protein stability, their potential role as drivers of cancer remains to be determined. Genes encoding members of the PRC2 complex are also mutated at elevated frequencies in particular cancers (Fig. 6). Intriguingly, these mutations can lead to either increased or decreased levels of H3K27me3, which varies depending on tissue or disease subtype (Conway et al. 2015). Below, we discuss instructive examples of how altered remodeler or Polycomb function promotes the development of cancer, highlighting our burgeoning understanding of the underlying molecular mechanisms. We start by reviewing rhabdoid tumors and synovial sarcoma in detail, because they are the best-understood cancers that are caused by aberant SWI/SNF function. Next, we review examples of cancers with additional SWI/SNF, NuRD, or PRC2 alterations. Finally, we discuss how perturbing the balance of SWI/SNF, NURD, and Polycomb activities may affect transcriptional programs and cell identity to promote oncogenesis.

## SMARCB1 in rhabdoid tumors

The first evidence for a causative role for mSWI/SNF defects in oncogenesis came from studies on rhabdoid tumors (RTs) (Versteege et al. 1998; Biegel et al. 1999). RTs are deadly pediatric cancers of the central nervous system (CNS), kidney and soft tissues, which typically occur in children <2 yr of age (Masliah-Planchon et al. 2015; Sredni and Tomita 2015; Frühwald et al. 2016). These cancers are also referred to as atypical teratoid RTs (ATRT) when located in the CNS or extracranial malignant RTs (ecMRT) when located elsewhere in the body. The vast majority of RT cases (∼99%) have biallelic loss of the *SMARCB1* gene (Fig. 6). A small minority of RTs are associated with mutations in *SMARCA4* rather than *SMARCB1* (Schneppenheim et al. 2010; Hasselblatt et al. 2014). Families harboring germline mutations in *SMARCB1* or *SMARCA4* are predisposed to the development of RT, referred to as RT predisposition syndrome (RTPS) (Sévenet et al. 1999; Sredni and Tomita 2015). The genomes of RTs display no substantial genomic instability and have very low mutational burden, with loss of *SMARCB1* as the sole recurring event (Lee et al. 2012; Lawrence et al. 2013; Chun et al. 2016; Torchia et al. 2016; Gröbner et al. 2018; Pinto et al. 2018). Thus, inactivation of *SMARCB1* appears to be sufficient to drive oncogenesis in the absence of collaborating genetic abnormalities. While biallelic loss of *Smarcb1* leads to early lethality during mouse development, haplo-insuffient mice are prone to develop tumors that resemble human RTs, showing loss of heterozygosity that is typical for a tumor suppressor (Klochendler-Yeivin et al. 2000; Roberts et al. 2000; Guidi et al. 2001). Reversible conditional inactivation of *Smarcb1* in mice revealed that, while it is essential for the survival of most normal cells, it also causes highly penetrant and aggressive cancers (Roberts et al. 2002). The vast majority of these tumors were T-cell lymphoma with only rare cancers that resembled RTs. Nonetheless, these mouse experiments established that loss of *Smarcb1* causes cancer in mouse models. Inactivation of *Smarcb1* at different stages of mouse development leads to dramatically different outcomes (Han et al. 2016). While full *Smarcb1* deletion during early development is lethal, its partial inactivation at embryonic day 6–10 (E6–E10) results in highly penetrant rapid onset CNS tumors that closely resemble human RT. In agreement with earlier observations, loss of *Smarcb1* in adult mice causes lymphomas instead of RT. The predisposition to the development of childhood RTs is not fully penetrant among RTPS families with germline mutations in *SMARCB1*, and its development in adults from these families is extremely rare (Taylor et al. 2000; Janson et al. 2006; Ammerlaan et al. 2008; Sredni and Tomita 2015). However, these adults frequently develop multiple schwannomas and benign tumors involving cranial and peripheral nerves. Thus, both patient data and mouse studies provide strong evidence for developmental stage as the major factor determining the consequences of *SMARCB1* inactivation.

While several studies have addressed the alterations in gene expression in RT; interpretations have been complicated by the substantial heterogeneity among tumors (Chun et al. 2016; Torchia et al. 2016; Richer et al. 2017; Pinto et al. 2018). An embryonic gene expression signature distinguishes RT from other cancers that are *SMARCB1*-deficient (Richer et al. 2017). Consistent with SWI/SNF's role as a member of the TrxG, dysregulation of Hox genes is frequently observed in RTs (Chun et al. 2016; Torchia et al. 2016; Richer et al. 2017; Pinto et al. 2018). Although more research is required to determine the significance of specific transcriptional perturbations in RT, the retention of an embryonic signature is likely a key aspect in the development of these tumors. Furthermore, loss of *SMARCB1* can affect cell cycle control in multiple ways. For example, inactivation of *SMARCB1* has been implicated in silencing of *CDKN2A*, encoding the pivotal tumor suppressor p16INK4a, in cell lines, mouse models, and human tumors (Betz et al. 2002; Oruetxebarria et al. 2004; Kia et al. 2008; Wilson et al. 2010; Venneti et al. 2011). *CDKN2A* is a well-characterized target of PcG proteins, linked to their ability to suppress cellular senescence (Jacobs et al. 1999; Bracken et al. 2007). Therefore, a key consequence of the loss of *SMARCB1* in RTs is that it compromises the ability of the SWI/SNF complex to counteract Polycomb-mediated repression of *CDKN2A* (Kia et al. 2008; Wilson et al. 2010).

The loss of SMARCB1 does not substantially debilitate the structural integrity of BAF or PBAF (Nakayama et al. 2017; Mashtalir et al. 2018) but does compromise their recruitment to key target sites (Kia et al. 2008; Tolstorukov et al. 2013; Nakayama et al. 2017; Wang et al. 2017). In addition, SMARCB1/Snf5 binds the SMARCA4/Snf2 catalytic subunit and stimulates nucleosome remodeling by mammalian and yeast SWI/SNF (Phelan et al. 1999; Sen et al. 2017). Recent genome-wide studies demonstrated that SMARCB1 is important for recruitment of SWI/SNF to developmental enhancers (Nakayama et al. 2017; Wang et al. 2017; Erkek et al. 2019). Loss of SWI/SNF binding at these sites is associated with PRC2 binding and repression of cognate developmental gene promoters, indicating that the SWI/SNF–Polycomb antagonism observed at the *CDKN2A* locus occurs genome wide. Importantly, EZH2 is essential for tumor formation after conditional loss of *SMARCB1* in mice (Wilson et al. 2010), and treatment with small molecule EZH2 inhibitors led to regression of RT in a xenograft model (Knutson et al. 2013). Thus, an important aspect of RT development is the loss of SWI/SNF antagonism to PcG repression. While the loss of SMARCB1 compromises both BAF and PBAF, it does not affect GBAF that lacks this subunit (Alpsoy and Dykhuizen 2018; Gatchalian et al. 2018; Mashtalir et al. 2018; Michel et al. 2018). Therefore, it is of interest that chemical probes that target the GBAF subunit BRD9 inhibit proliferation of RT cell lines (Krämer et al. 2017; Michel et al. 2018). Collectively, these observations suggest that RTs might be especially vulnerable to combined targeting of BRD9 and EZH2. In addition, due to their stable genomes, the canonical tumor suppressor pathways remained intact in RT, and might be exploited as well. In conclusion, the detailed dissection of gene control in RT starts to provide leads for potential therapeutic intervention.

## SS18-SSX fusions in synovial sarcoma

Synovial sarcoma is an aggressive, poorly differentiated malignancy that can occur in patients of all ages but is particularly common in children and young adults with a peak incidence between 20 and 30 yr of age. The hallmark genetic abnormality is a chromosomal translocation involving chromosomes X and 18, t(X;18). This rearrangement fuses the N terminus of the BAF and GBAF subunit SS18 (also known as SYT) to the C terminus of one of three related proteins, SSX1, SSX2, or SSX4 (Clark et al. 1994; de Leeuw et al. 1995; Skytting et al. 1999). Like RTs [other than t(X:18)], synovial sarcoma tumors contain few, if any, genetic abnormalities (Barretina et al. 2010; Abeshouse et al. 2017). This suggests that the SS18-SSX fusion protein is the primary driver of disease development. Indeed, conditional expression of SS18-SSX leads to the development of a synovial sarcoma-like disease in mice (Haldar et al. 2007). Similar to results observed in *Smarcb1*-deficient mouse models, both cellular context and developmental timing are critical for the ability of SS18-SSX to induce tumors. For example, while the expression of SS18-SSX in mature muscle lineages causes myopathy without tumorigenesis, when expressed in immature muscle progenitor cells, adult mice develop aggressive tumors with 100% penetrance (Haldar et al. 2007).

SS18-SSX dominantly assembles into SWI/SNF, causing the eviction and proteasomal degradation of both the wild-type SS18 and SMARCB1 (Kadoch and Crabtree 2013). Therefore, in both RT and synovial sarcoma, SMARCB1 is lacking from BAF (but not from PBAF). However, the assembly of SS18-SSX into BAF, and not SMARCB1 loss, appears to be the primary pathogenic alteration (McBride et al. 2018). SS18-SSX does not associate with PBAF, but it assembles into both BAF and GBAF (Brien et al. 2018; McBride et al. 2018). Given their compositional differences, it is likely that incorporation of SS18-SSX has unique functional effects as part of BAF versus GBAF. These differences need to be explored given the recent demonstration that synovial sarcoma cells are exquisitely dependent on GBAF function (Brien et al. 2018; Michel et al. 2018). Depletion of SS18-SSX correlates with increased PRC2 mediated H3K27me3 and repression of many SS18-SSX target genes (Banito et al. 2018; McBride et al. 2018). SS18-SSX containing BAF and GBAF appear to be more potent antagonists of Polycomb function than their wild-type counterparts (Kadoch and Crabtree 2013). This suggests that SS18-SSX containing complexes may act dominantly to aberrantly activate Polycomb target genes. Consistent with this notion, SS18-SSX activates the expression of many developmental regulators (Banito et al. 2018; McBride et al. 2018). Moreover, cells expressing SS18-SSX appear to be blocked in their capacity to differentiate (Haldar et al. 2007). Depletion of SS18-SSX triggers downregulation of developmental gene expression signatures and differentiation toward a mesenchymal cell fate (Banito et al. 2018; Brien et al. 2018). It has also been suggested that SS18-SSX associates with KDM2B, which may lead to its mis-direction to KDM2B-bound loci (Banito et al. 2018). Collectively, these results suggest that SS18-SSX containing BAF and GBAF oppose Polycomb mediated repression of a primitive development transcriptional program, causing a block to cellular differentiation.

## Mutations of SWI/SNF genes in multiple other cancer types

Studies on RT and synovial sarcomas established that defects in SWI/SNF subunits SMARCB1 and SS18 cause cancer. Sequencing studies revealed that the genes encoding additional SWI/SNF subunits are frequently mutated in a wide array of other cancer types (Fig. 6). Here, we restrict our discussion to a selection of SWI/SNF subunits for which functional studies are beginning to provide mechanistic insights. The BAF-specific *ARID1A* gene is the most commonly mutated SWI/SNF component in cancer (Fig. 6). Its function is primarily lost due to truncating mutations as has been reported in multiple cancers, including bladder cancer (Gui et al. 2011), uterine endometrial carcinoma (Kandoth et al. 2013), and neuroblastoma (Sausen et al. 2013). Moreover, recent pan-cancer analyses of almost 10,000 adult and 1600 pediatric cancers further demonstrate the significance of *ARID1A* mutations in multiple malignancies (Bailey et al. 2018; Gröbner et al. 2018; Ma et al. 2018). While our mechanistic insights on the effects of ARID1A loss are still limited, recent studies suggested that impaired enhancer function might play a central role. In a mouse model of colon cancer, based on the conditional deletion of *Arid1a*, loss of ARID1A correlated with reduced binding of SWI/SNF at enhancers and reduced expression of cognate genes (Mathur et al. 2017). Another study established that ARID1A loss leads to reduced chromatin accessibility at enhancers, impeding transcription factor binding (Kelso et al. 2017). Consistent with the observed reductions in gene expression and chromatin accessibility, many of these enhancers had reduced levels of H3K27ac (Kelso et al. 2017; Mathur et al. 2017). Furthermore, the deletion of *Arid1a* in a mouse model of hepatocellular cancer also leads to a reduction in chromatin accessibility and associated gene expression (Sun et al. 2017). Truncating mutations of ARID1A abrogate its ability to associate with the core of the SWI/SNF complex (Mashtalir et al. 2018). Thus, like its *Drosophila* homolog Osa (Moshkin et al. 2012), loss of ARID1a impairs BAF recruitment to chromatin. Given the reductions in both chromatin accessibility and H3K27Ac at enhancers following loss of ARID1a, it is tempting to speculate that the absence of BAF allows NuRD to promote repressive chromatin at these loci. This could contribute to an impaired ability of these cancer cells to activate key genes required for differentiation. Finally, oncogene-induced senescence and activation of p53 in hepatocellular carcinoma cells depends on ARID1B/BAF (Tordella et al. 2016).

PBRM1 loss-of-function genetic alterations are present in ∼40% of kidney renal clear cell carcinoma (KIRC), making it second to the VHL tumor suppressor in terms of frequency (Fig. 6; Varela et al. 2011). Gene expression analysis revealed substantial changes due to loss of PBAF function in these cells, which correlates with responsiveness to immune checkpoint therapy (Miao et al. 2018). In an independent screen to identify barriers to killing of cancer cells by cytotoxic T cells, inactivation of Pbrm1, Arid2 and Brd7 was found to mediate resistance (Pan et al., 2018). Both studies suggest that PBAF control of interferon-stimulated gene expression promotes immune resistance. Loss of PBAF function increased tumor cell secretion of chemokines that recruit effector T cells. Thus, targeting PBAF might sensitize cancer cells to immunotherapy. Paradoxically, in clear cell renal cell carcinoma, loss of PBAF function might promote both tumorigenicity and susceptibility to antitumor immunity.

SMARCA4 is the most commonly mutated Snf2-like ATPase in cancer, including several adult and pediatric malignancies (Fig. 6; Bailey et al. 2018; Gao et al. 2018; Gröbner et al. 2018; Hodges et al. 2018). Similar to ARID1A, many SMARCA4 mutations are predicted to inactivate, suggesting that it functions as a tumor suppressor. A portion of the cancer-associated SMARCA4 mutations occur within the ATPase domain (Hodges et al. 2018). Introduction of these SMARCA4 ATPase mutations into mouse ESCs leads to a loss of chromatin accessibility and H3K27Ac at enhancers, accompanied by transcriptional down-regulation, and accumulation of Polycomb and H3K27me3 at the promoters of associated genes (Stanton et al. 2017; Hodges et al. 2018). However, as observed for SMARCB1-deficient RT cells, increased Polycomb activity occurs specifically at gene promoters and not at enhancers. These observations indicate that loss of SWI/SNF function in cancer leads to alterations in enhancer landscapes. We speculate that this might be due to increased NuRD activity at these sites. The reduced activity of SWI/SNF-dependent enhancers correlates with an increased activity of Polycomb at the associated gene promoters, and attenuated transcription. These effects appear to alter the developmental potential of SWI/SNF mutant cells, making them linger in an undifferentiated (or incompletely differentiated) state. A recent analysis of somatic histone mutations in cancer revealed a substantial number of alterations in the globular domain that have been implicated in remodeler function (Nacev et al., 2019). These include mutations homologous to yeast mutants that alleviate the need for SWI/SNF, and alterations in the acidic patch (Fig. 3C) that are predicted to impair remodeling by ISWI.

## The complex roles of NuRD in human cancer

In addition to its well-established roles in cell differentiation programs, disruption of NuRD function has also been implicated in oncogenesis. However, cancer-associated mutations in NuRD subunits occur less frequently than in SWI/SNF components (Kadoch et al. 2013; Bailey et al. 2018; http://www.cbioportal.org). The gene encoding the CHD5 member of NuRD is a tumor suppressor that is frequently deleted in high-risk neuroblastoma and glioma (Bagchi et al. 2007). It is expressed in normal neuronal tissues and in low-risk neuroblastomas, but its levels are reduced in tumors from high-risk neuroblastoma patients (Fujita et al. 2008; Garcia et al. 2010; Egan et al. 2013). CHD5 is required for terminal neuronal differentiation and has a dual role in facilitating the activation of neuronal genes, as well as the repression of a cohort of Polycomb target genes (Egan et al. 2013). Therefore, its loss in neuroblastoma has been proposed to impede the ability of neural cells to undergo terminal differentiation. The role of CHD4 in human cancer is more complicated. In some cancers, CHD4 is mutated with increased frequency, but compelling evidence that these are driver mutations remains lacking. Moroever, CHD4 has also been linked with pro-oncogenic functions. The recruitment of CHD4-NuRD by the DNA-binding transcription factor ZFHX4 is crucial for the maintenance of therapy-resistant tumor-initiating cells in glioblastoma (Chudnovsky et al. 2014). CHD4 is required for the maintenance of MLL-AF9 rearranged acute myeloid leukemia cells but not for growth of normal hematopoietic cells (Heshmati et al. 2018). Conversely, MBD3-CHD4-NuRD prevents tumorigenesis by constraining a B-cell transcriptional program through restriction of lineage-specific enhancers and promoters (Loughran et al. 2017). This process involves opposing activities of NuRD and SWI/SNF, again emphasizing the importance of chromatin balance in developmental transcription control. The DOC1 subunit of NuRD is lost in the majority of oral cancers and correlates with tumor invasion and adverse outcomes (Shintani et al. 2001; Mohd-Sarip et al. 2017). The loss of DOC1 affects recruitment of NuRD to a selection of genes that control proliferation and EMT (Mohd-Sarip et al. 2017). At these loci, NuRD and SWI/SNF compete for binding and generate opposite chromatin states. SWI/SNF drives formation of open chromatin and counters Polycomb binding. In contrast, NuRD mediates nucleosome invasion, histone deacetylation, recruitment of Polycomb and KDM1A, and transcriptional repression. Although not as well explored as the connection between SWI/SNF and cancer, these studies indicate that alterations in NuRD integrity can contribute to oncogenesis. Mutations in or deregulation of NuRD can disturb the balance with SWI/SNF, leading to the corruption of developmental programs and proliferation control. As for SWI/SNF, subunit-dependent gene selectivity is likely to explain the association with specific types of cancer.

## Deregulation of Polycomb function in cancer

Initial links between deregulation of Polycomb function and cancer were related to their overexpression rather than driver mutations (Pasini and Di Croce, 2016). However, there is little evidence of driver mutations in genes encoding subunits of cPRC1 in human cancer. So, while overexpression of PRC1 components such as PCGF4/BMI1 might promote oncogenesis, it might also be a consequence of the proportion proliferating or stem like cells in the tumor, rather than a cause. This limited evidence of cancer driver mutations in genes encoding cPRC1 subunits might be related to the high level of redundancy among cPRC1 subunits, which would limit the impact of mutations. However, somatic mutations in the ncPRC1 subunit BCOR have been identified in AML and myelodysplastic syndrome, and mouse studies support a tumor suppressor function in leukemia (Damm et al. 2013; Tanaka et al. 2017; Kelly et al. 2019). Bcor has a key function in the regulation of the hematopoietic stem cell transcription network and its inactivation drives expansion of myeloid progenitor cells and cooperates with oncogenic Kras to drive the development of leukemia (Kelly et al. 2019). Thus, while the roles of some PRC1 components in cancer seems mostly related to their requirement for stem cell survival, BCOR appears to be a bona fide tumor suppressor.

Multiple cancer genome sequencing studies have revealed that the function of PRC2 is disrupted on a genetic level in a wide array of cancers, leading to both loss and gain of activity (Conway et al. 2015). Prior to these studies, EZH2 was reported to be highly expressed in multiple cancers as a result of being an E2F-regulated gene, downstream from the pRB pathway (Bracken et al. 2003). However, elevated EZH2 expression is not considered to lead to deregulation of H3K27 methylations, because the stoichiometry of the core PRC2 complex would require correspondingly high levels of the EED and SUZ12 subunits (Wassef et al. 2015). In 2010, recurrent heterozygous mutations of the *EZH2* catalytic SET domain in 22% of Diffuse large B-cell lymphomas (DLBCLs) and 10% of follicular lymphomas (FLs) were reported for the first time (Fig. 6; Morin et al. 2010). These mutations were originally thought to induce loss-of-function effects on EZH2 enzymatic activity; however, additional work demonstrated that the mutant EZH2 has altered substrate preferences (Sneeringer et al. 2010). The preferred activity of wild-type EZH2 is the conversion of H3K27me0 to me1 and H3K27me1 to H3K27me2, while the enzyme is relatively inefficient at the final conversion step to H3K27me3. Owing to changes in substrate-binding modality, lymphoma associated EZH2 SET domain mutants have an enhanced ability to convert H3K27me2 to me3. As a result, these “change-of-function” EZH2 mutants cooperate with the wild-type enzyme, pushing the kinetics of PRC2 activity toward increased H3K27me3 production. This leads to aberrantly high global levels of H3K27me3, with concomitant reductions of H3K27me2 levels in EZH2 mutant lymphoma cells. Importantly, in a mouse model, conditional expression of this “change-of-function” EZH2 mutant in B-cell lineages replicates these H3K27 methylation changes and, while augmented by overexpression of BCL2 or loss of p53, is sufficient to cause malignancy (Béguelin et al. 2013; Souroullas et al. 2016).

Our understanding of the molecular mechanisms underlying oncogenesis in these EZH2 mutant contexts is still limited. ChIP-seq (chromatin immunoprecipitation [ChIP] combined with high-throughput sequencing) experiments have suggested that increased levels of H3K27me3 mark the promoter regions of genes required for cell cycle exit and terminal B-cell differentiation (Béguelin et al. 2013; Souroullas et al. 2016). This correlates with reduced expression of many of these genes, suggesting that mutant EZH2 impedes the differentiation potential of immature B-cells. Moreover, cPRC1 appears to maintain the repression of these genes, which may be linked to recruitment of the CBX8 containing cPRC1 via binding to H3K27me3 at these sites (Béguelin et al. 2016). To date, the consequences of reduced H3K27me2 levels in these cells and its functional relationship with H3K27me3 have yet to be examined. This might be important given that H3K27me2 is known to mark up to 70% of all histone H3 in normal cells (Ferrari et al. 2014). Moreover, H3K27me2 has been suggested to perform repressive functions at intergenic sites that are likely altered in EZH2 mutant lymphoma cells (Ferrari et al. 2014; Conway et al. 2015).

In contrast to lymphomas, the genes encoding core PRC2 subunits are tumor suppressors in other cancer types. For example, in pediatric T-cell acute lymphoblastic leukemias (T-ALLs) and malignant peripheral nerve sheath tumors (MPNSTs) the *EZH2*, *EED*, and *SUZ12* genes are subject to deletions or inactivating mutations (Ntziachristos et al. 2012; Zhang et al. 2012; Lee et al. 2014). These loss-of-function alleles are associated with reductions in the global levels of both H3K27me2 and H3K27me3. Functional studies showed that the loss of PRC2 activity in T-ALL and MPNST leads to aberrant activation of some PRC2 target genes (Ntziachristos et al. 2012; Lee et al. 2014). It is likely that the loss of PRC2-mediated H3K27me3 at these genes leads to increased activity of SWI/SNF chromatin remodellers. The function of PRC2 is also deregulated in pediatric diffuse intrinsic pontine gliomas (DIPGs), a highly aggressive pediatric brainstem tumor (Mohammad and Helin 2017). The discovery that up to 80% of DIPG have mutations in two genes encoding histone H3F3A (H3.3) or HIST3H1B (H3.1) led to the finding of global reductions in H3K27me2 and H3K27me3 in these cells (Conway et al. 2015). Subsequent work has suggested that H3K27M can act as a dominant-negative mutation that, at least partially, inhibits PRC2 (Mohammad and Helin 2017).

Taken together, it is clear that the function of PRC2 is deregulated in several types of cancer, accompanied by changes in the global levels and genomic distributions of H3K27me2 and H3K27me3. While it remains unclear how these epigenomic changes contribute to the development of cancer, it is likely that they confer context-dependent blocks to cellular differentiation and increased vulnerability to aberrant cancer signaling pathways.

## Therapeutic opportunities: restoring or tipping the chromatin balance

**Figure 7.**
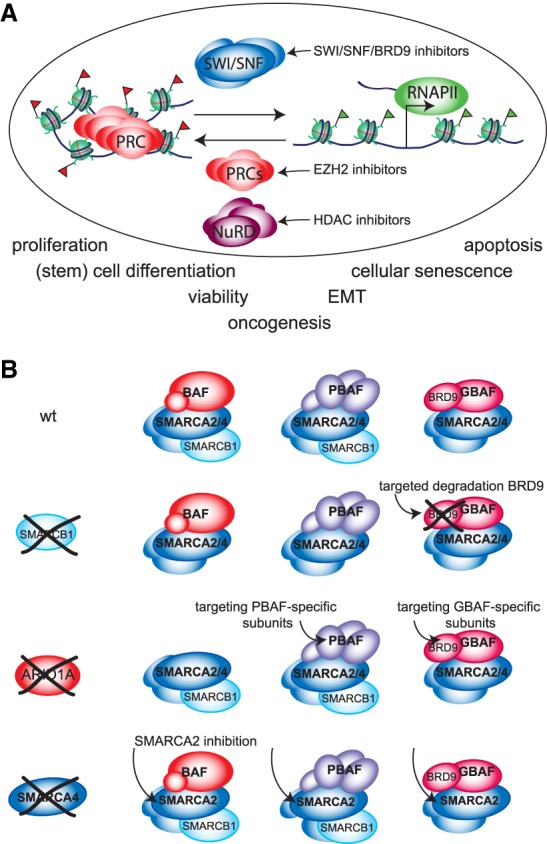
Therapeutic opportunities. (*A*) Maintaining chromatin equilibrium. Physiological gene expression depends on the balanced interplay between SWI/SNF, NuRD, and Polycomb. A disturbance in this chromatin equilibrium—for example, due to loss of one of the SWI/SNF subunits—can promote oncogenesis due to misexpression of genes that regulate cell proliferation, cell differentiation, EMT, cellular senescence, or apoptosis. A therapeutic strategy might involve restoring the chromatin balance by compensating for loss of SWI/SNF function by inhibition of PRC2 or NuRD. Conversely, loss of Polycomb function might be compensated for by inhibition of SWI/SNF. (*B*) Tipping the chromatin balance. Synthetic lethality provides another potential therapeutic strategy for cancers with loss-of-function mutations in SWI/SNF. The residual SWI/SNF complexes are often essential for the viability of these tumor cells, and therefore present attractive therapeutic targets. For example, SMARCB1 mutant RT cells depend on BRD9 function. Alternatively, loss of ARID1A might create a crucial requirement for PBAF or GBAF. The loss of one subunit (e.g., SMARCA4 in lung cancer cells) can also create a crucial dependency on its paralog, SMARCA2. Consequently, targeting paralog function in these settings may provide an effective therapeutic option. For details and references, see the text.

Normal gene expression control depends on the equilibrium between SWI/SNF opposed by NuRD and Polycomb (Fig. 7A). As discussed in this review, many cancers have a disturbance of this chromatin balance. This common feature might be exploited therapeutically. For example, in the context of loss of SWI/SNF function, unrestrained Polycomb activity appears to mediate aberrant repression of transcriptional programs related to cellular differentiation and tumor suppression. Consequently, targeting PRC2 function in SWI/SNF mutant cancers may reverse the oncogenic chromatin changes that underlie disease development. Consistent with this, codeletion of *Ezh2* and *Snf5* is not compatible with cancer development in mice (Wilson et al. 2010). The recent development of small-molecule EZH2 methyltransferase inhibitors has provided an opportunity to examine the clinical utility of PRC2 inhibition in multiple cancers, including those carrying inactivating mutations in genes encoding SWI/SNF subunits (McCabe et al. 2012; Knutson et al. 2013; Campbell et al. 2015). As a proof of concept, SMARCB1-deficient RT cells are more sensitive to treatment with EZH2 inhibitors than wild-type cells (Knutson et al. 2013). Moreover, EZH2 inhibition blocks the progression of SMARCB1 mutant cancers in vivo, which has motivated the establishment of ongoing clinical trials with EZH2 inhibitors in RTs. Early results have reported clinical responses in a subset of SMARCB1 negative patients (Italiano et al. 2018). EZH2 inhibitor treatment of non-small cell lung cancer cells bearing SMARCA4 mutations sensitizes these cells to chemotherapy, an effect that is not apparent in SMARCA4 wild-type cells (Fillmore et al. 2015). Furthermore, *Arid1a* mutant, but not wild-type, ovarian cancer cell lines are also sensitive to reduction of EZH2 (Bitler et al. 2015; Kim et al. 2015). These findings indicate that targeting PRC2 activity in SWI/SNF mutant cancers may provide an effective means to treat this large cohort of patients. Finally, targeting HDAC activity, particularly HDAC2, blocks the progression of *Arid1a* mutant ovarian cancer cells (Fukumoto et al. 2018). Thus, NuRD complexes, harboring HDAC1/2, may also be relevant in SWI/SNF mutant cancers. However, the general efficacy of HDAC inhibitors remains to be systematically examined in SWI/SNF mutant contexts.

Accumulating evidence indicates that the SWI/SNF complexes themselves may be an effective therapeutic target in SWI/SNF mutant cancers. For example, GBAF is essential for the continued growth and survival of synovial sarcoma cells (Brien et al. 2018; Michel et al. 2018). The selective targeting of the GBAF subunit BRD9 in a mouse model of synovial sarcoma effectively blocked tumor progression, supporting the therapeutic promise of this approach. Synthetic lethality is another potential strategy for cancers with loss-of-function mutations in genes encoding SWI/SNF subunits (Helming et al. 2014; Hohmann and Vakoc 2014). Typically, the residual SWI/SNF complexes are essential in these cancer cells, which may be exploited therapeutically (Fig. 7B). For example, SMARCB1 deficient cells require residual SWI/SNF function, because concomitant loss of SMARCB1 and SMARCA4 blocks tumor development (Wang et al. 2009). Moreover, SMARCB1 mutant RT cells also depend on BRD9 function (Krämer et al. 2017; Michel et al. 2018). ARID1A mutant cancer cells often require the function of its paralog ARID1B (Helming et al. 2014; Kelso et al. 2017; McDonald et al. 2017). Likewise, SMARCA4 mutant lung cancer cells depend on SMARCA2 for their continued growth and survival (Wilson et al. 2014; McDonald et al. 2017). Thus, targeting paralog function in these settings may provide an effective therapeutic option (Fig. 7B). Recent drug development efforts are beginning to uncover molecules that specifically target SWI/SNF subunits such as ARID1A, BRD7, and BRD9, which will allow this idea to be tested (Hohmann et al. 2016; Theodoulou et al. 2016; Remillard et al. 2017; Brien et al. 2018; Marian et al. 2018).

The mutation of EZH2 in B-cell lymphomas motivated the development of small-molecule inhibitors that act as SAM-competitive binders of the SET domain and exhibit high selectivity for EZH1/2 (Helin and Dhanak 2013; Kim and Roberts 2016). These compounds are capable of reducing global levels of H3K27me1/2/3 in cells, regardless of their EZH2 mutational status. Nevertheless, EZH2 “change-of-function” mutant cells are particularly sensitive to these drugs. This motivated the establishment of clinical trials to examine their effects in lymphoma patients (Brien et al. 2016; Kim and Roberts 2016). Recent reporting on these ongoing clinical studies indicated significant responses in EZH2 mutant patients, with as many as 92% of EZH2-mutant follicular lymphoma patients responding to treatment (Italiano et al. 2018). Functional studies indicated that EZH2 inhibition leads to reductions in global H3K27me3 levels, which is associated with up-regulation of PRC2 target genes (McCabe et al. 2012). A complicating factor is the identification of secondary mutations in both wild-type and mutant EZH2 alleles in B-Cell lymphoma cells lines, which can cooperate to confer resistance to EZH2 inhibitors (Baker et al. 2015; Gibaja et al. 2016). This emphasizes the probability that acquired resistance to EZH2 inhibitors will also occur in patients. Thus, alternative targets to disrupt both wild-type and mutant EZH2 in cancer will be needed. The development of potent EED inhibitors, which block its ability to allosterically activate PRC2 via its engagement with H3K27me3, might help to bypass this complication (He et al. 2017; Qi et al. 2017). Importantly, cell lines that had developed resistance to EZH2 SET domain inhibitors remained sensitive to this EED inhibitor, indicating that they may help to circumvent primary acquired resistance. However, an alternative strategy may be needed to treat patients with T-ALL and MPNST that harbor loss-of-function mutations in PRC2. In the context of the chromatin balance paradigm (Fig. 7A), it is tempting to speculate that specific inhibitors of SWI/SNF might open up new opportunities for treatment of these cancer types. In conclusion, the detailed knowledge of the molecular mechanisms and biology of chromatin remodelers and Polycombs, is now enabling the development of promising new therapeutic approaches to treat many human cancer types.
